# Evolving evidence in the treatment of primary and recurrent posterior cruciate ligament injuries, part 2: surgical techniques, outcomes and rehabilitation

**DOI:** 10.1007/s00167-020-06337-2

**Published:** 2020-10-30

**Authors:** Philipp W. Winkler, Bálint Zsidai, Nyaluma N. Wagala, Jonathan D. Hughes, Alexandra Horvath, Eric Hamrin Senorski, Kristian Samuelsson, Volker Musahl

**Affiliations:** 1 Department for Orthopaedic Sports Medicine, Klinikum Rechts der Isar, Technical University of Munich, Ismaninger Str. 22, 81675 Munich, Germany; 2 Department of Orthopaedic Surgery, UPMC Freddie Fu Sports Medicine Center, University of Pittsburgh, 3200 S. Water St., Pittsburgh, PA 15203 USA; 3 Department of Orthopaedics, Sahlgrenska University Hospital, Mölndal, Sweden; 4 Department of Internal Medicine and Clinical Nutrition, Institute of Medicine, Sahlgrenska Academy, University of Gothenburg, Gothenburg, Sweden; 5 Department of Health and Rehabilitation, Institute of Neuroscience and Physiology, Sahlgrenska Academy, University of Gothenburg, Gothenburg, Sweden; 6 Department of Orthopaedics, Institute of Clinical Sciences, Sahlgrenska Academy, University of Gothenburg, Gothenburg, Sweden

**Keywords:** Posterior cruciate ligament, PCL, Revision, Failure, Knee, Single-bundle, Double-bundle, Risk factors, Transtibial, Tibial inlay

## Abstract

**Abstract:**

Isolated and combined posterior cruciate ligament (PCL) injuries are associated with severe limitations in daily, professional, and sports activities as well as with devastating long-term effects for the knee joint. As the number of primary and recurrent PCL injuries increases, so does the body of literature, with high-quality evidence evolving in recent years. However, the debate about the ideal treatment approach such as; operative vs. non-operative; single-bundle vs. double-bundle reconstruction; transtibial vs. tibial inlay technique, continues. Ultimately, the goal in the treatment of PCL injuries is restoring native knee kinematics and preventing residual posterior and combined rotatory knee laxity through an individualized approach. Certain demographic, anatomical, and surgical risk factors for failures in operative treatment have been identified. Failures after PCL reconstruction are increasing, confronting the treating surgeon with challenges including the need for revision PCL reconstruction. Part 2 of the evidence-based update on the management of primary and recurrent PCL injuries will summarize the outcomes of operative and non-operative treatment including indications, surgical techniques, complications, and risk factors for recurrent PCL deficiency. This paper aims to support surgeons in decision-making for the treatment of PCL injuries by systematically evaluating underlying risk factors, thus preventing postoperative complications and recurrent knee laxity.

**Level of evidence:**

V.

## Introduction

Posterior cruciate ligament reconstruction (PCL-R) techniques have been studied and evolved over the past decades, providing a solid and evidence-based foundation for the operative management of posterior cruciate ligament (PCL) injuries [1, 39, 41, 45, 77, 88]. Single-bundle (SB) and double-bundle (DB) PCL-R based on the transtibial or tibial inlay technique and implemented by an open, arthroscopically assisted, or all-arthroscopic approach, all offer certain advantages and disadvantages [1, 8, 15, 16, 34, 40, 45, 75, 77, 87, 89]. On the other hand, the PCL is characterized by a strong intrinsic healing capability, making the non-operative treatment approach a viable option, especially for partial PCL tears and tibial avulsion injuries of the PCL [2, 3, 26, 71, 72]. Good subjective and objective long-term outcomes after non-operative treatment with a fairly low prevalence of 11% of moderate to severe osteoarthritis (OA) after more than 14 years follow-up, keep the debate about the optimal treatment approach, operative vs. non-operative, in PCL injured patients ongoing [71]. As a result, a wide range of viable treatment options is available, enabling an individualized treatment approach based on the injury pattern and the patient’s compliance and demands.

In this review, we present the indications, techniques, and outcomes of operative and non-operative treatment of primary and recurrent PCL injuries. Moreover, risk factors associated with recurrent PCL deficiency and future perspectives are outlined.

## Indications for PCL-R

Operative treatment of PCL injuries is indicated for patients with symptomatic grade III (complete) tears displaying inadequate functional improvement in response to non-operative treatment. Furthermore, patients with PCL injuries with high-grade knee laxity, or combined with intraarticular or capsuloligamentous injuries should be considered for operative treatment [55, 79]. A side-to-side difference in posterior tibial translation (PTT) greater than 8 mm revealed by stress-radiography indicates a complete PCL tear and presents an indication for operative treatment for symptomatic patients [29, 68, 69]. Additionally, the patient’s demands are essential in treatment decision-making, leading to the recommendation of PCL-R in an athletic population [39]. There is currently insufficient evidence in the literature to support a definitive treatment protocol; however, the historical preference towards non-operative treatment of isolated PCL injuries has recently displayed a shift in favor of operative treatment [17, 61, 79]. Indications for operative treatment may be based on increased translational and rotational tibial movement compared to contralateral PCL intact knees (as measured on in vivo kinematic analyses) [43]. An increasing number of intra-articular injuries (meniscus, cartilage), pathological changes of the anterior cruciate ligament (ACL), and an increased joint contact pressure (tibiofemoral and patellofemoral) have been observed in PCL deficient patients [20].

A failure rate of 1–25% after primary PCL-R is reported, mounting to 45% if unfavorable patient-reported outcomes (Knee Injury and Osteoarthritis Outcome Score < 40 points) are considered as subjective failure [4, 36, 41, 46, 81, 88]. However, inadequate reporting and varying definitions of failures require caution in the interpretation of failure rates. Debilitating pain and functional impairment during daily activities combined with a PTT of  ≥ 10 mm or confirmed graft failure based on magnetic resonance imaging (MRI) scans are accepted indications for revision PCL-R [58, 59]. The aim of revision PCL-R is to address the cause of initial failure and eliminate concurrent pathological changes causing pain and instability [41, 59]. Therefore, in case of revision PCL-R, extended diagnostic work-up including MRI, hip-knee-ankle radiographs, and computed tomography (CT) scans are required to assess concomitant injuries, lower limb alignment, and prior bone tunnel placement. The requirement of bone grafting due to prior semi-anatomic bone tunnels or the need for corrective osteotomies sometimes require staged revision PCL-R.

## Technical aspects in PCL-R

**Fig. 1:**
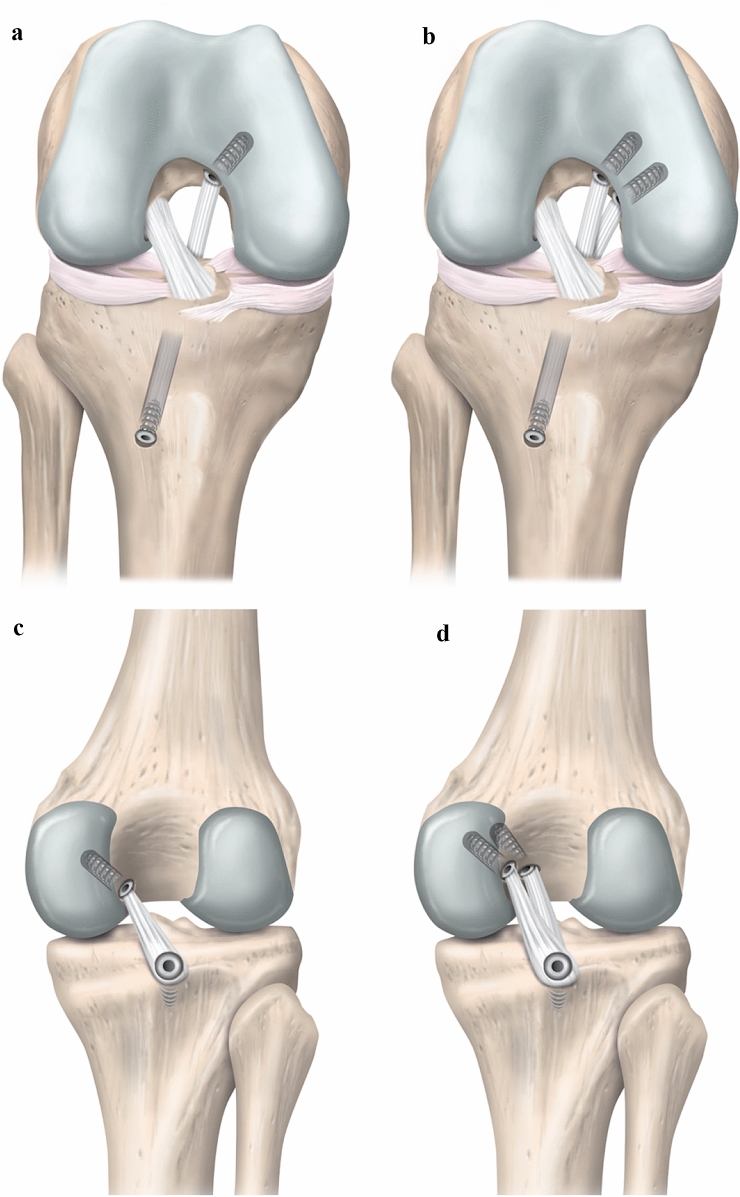
Schematic illustration of the transtibial and tibial inlay posterior cruciate ligament reconstruction techniques. Single-bundle (**a**) and double-bundle (**b**) transtibial technique (right knee, anterior view). Single-bundle (**c**) and double-bundle (**d**) tibial inlay technique (right knee, posterior view)

Owing to the anatomy of the PCL and the complex nature of PCL injuries, there is no consensus for a specific operative technique when considering PCL-R. Many biomechanical and clinical research efforts have enabled the development of techniques focusing on anatomic restoration of native knee kinematics [28, 66, 85]. Variations between the different reconstructive techniques depend primarily on tunnel placement, graft choice, graft positioning and fixation, and the choice of an arthroscopic or open surgical approach (Fig. 1). While studies have reported that both SB and DB PCL-R improve knee kinematics, biomechanical results have recently shown DB PCL-R to closer approximate the native state of the ligament [15, 23, 85].

The focus of anatomic SB PCL-R is to restore knee kinematics by aligning the path of the PCL graft with the native course of the stronger and more prominent anterolateral bundle (ALB). On the other hand, DB PCL-R aims to provide an alternative method by restoring the synergistic functionality of the ALB and posteromedial bundle (PMB) to closer approximate the native anatomy [5, 79].

Tibial graft fixation during anatomic SB and DB PCL-R can be performed using the transtibial or the tibial inlay technique by an all-arthroscopic, arthroscopic assisted, or an open approach [8, 42, 62, 76]. Positioning of a PCL drill guide medial to the tibial tubercle just proximal to the pes anserinus, aiming for a sagittal angle of 45°, allows subsequent insertion of a guidewire to reach the anatomic tibial PCL insertion zone when performing the transtibial technique (a more detailed description of radiographic landmarks for the native femoral and tibial insertion zones of the PCL is given in Part 1 of the evidence-based update on the management of primary and recurrent PCL injuries) [18, 74]. A frequently reported drawback of the transtibial technique is the formation of an acute angle by the PCL graft exiting the tibial tunnel, also known as the “killer turn” [53]. Graft degeneration, abrasion, and delayed or incomplete graft maturation are attributed to the killer-turn effect, which is believed to cause residual posterior laxity and increase the failure rate after transtibial PCL-R [8, 39, 84]. Protection of the posterior neurovascular bundle is of utmost importance. One study has shown that the mean distance between the popliteal artery and the posterior tibial cortex 5 mm distal to the joint line was significantly greater at 90° compared to 0° of knee flexion (7.7 ± 3.8 mm vs. 1.6 ± 1.3 mm) [33]. Accordingly, tibial tunnel drilling with fluoroscopic guidance is recommended at 90° knee flexion to prevent injury of the neurovascular structures.

The tibial inlay techniques have been developed to facilitate graft fixation by employing bone troughs for tibial graft insertion and thereby restoring the original PCL anatomy while diminishing PCL graft stress by avoiding the killer-turn effect [8]. The open or arthroscopically assisted approach to the tibial inlay technique requires the patient to be positioned in the lateral decubitus or prone position, or in supine position if the hip and knee are freely movable. After careful dissection, the bone block of the graft can be placed in the created trough and a screw is used for tibial graft fixation [8, 42].

Posterior cruciate ligament graft fixation is usually performed using interference screws, or suspensory fixation techniques [14, 21, 60, 79]. To optimize graft loads and to restore native knee kinematics, the ideal knee flexion angle during graft fixation is important [24, 31, 32]. Consequently, it has been demonstrated that graft fixation angles ranging from 75° to 105° of knee flexion restore knee kinematics to the same extent after SB PCL-R, albeit failing to restore laxity compared to the intact state [31]. In the case of DB PCL-R, PMB graft fixation at 0° and ALB graft fixation at 90° or 105° of knee flexion best restores native knee kinematics while avoiding excessive restriction of the tibiofemoral joint [32].

Graft choice is crucial in PCL-R and includes allografts and autografts as well as soft tissue-only, bone-tendon, and bone-tendon-bone grafts. While soft tissue-only grafts are often preferred for the transtibial technique, tendon-bone grafts are usually used when performing the tibial inlay technique [8, 42, 67]. Less residual PTT has been demonstrated when using autografts compared to allografts for PCL-R [6, 7]. Although a statistically significant difference between autograft and allograft use was demonstrated, the clinical relevance of a mean side-to-side difference in PTT of less than 1.5 mm has to be questioned [6, 7, 44, 80]. The use of allografts was associated with a shorter operation time, while autografts were associated with donor-site morbidity. With respect to patient-reported outcomes and graft failure rates, no difference between allografts and autografts for PCL-R could be demonstrated [6, 7]. Therefore, no appreciable difference seems to exist between the usage of allogenous or autologous tissue for PCL-R [25, 46, 82].

## Clinical outcomes following PCL-R

### Single-bundle (SB) vs. double-bundle (DB)

Biomechanical studies have demonstrated that only anatomic DB PCL-R is able to restore native knee laxity across the entire range-of-motion and that SB PCL-R leads to residual posterior laxity [31, 32, 66]. Consequently, several clinical studies have advocated DB PCL-R and have demonstrated improved clinical and functional outcomes compared to SB PCL-R [15, 36, 45]. A randomized controlled trial comparing isolated SB (*n* = 22) and DB (*n* = 24) allograft PCL-R reported significant improvements in patient-reported outcomes (International Knee Documentation Committee (IKDC) Subjective Knee Form, Lysholm Score, Tegner Activity Scale) and reduced knee laxity (KT-1000) for both techniques with a minimum of two-years of follow-up [45]. The DB group showed superior results in knee laxity as well as objective and subjective IKDC compared to the SB group [45]. Another randomized controlled trial showed similar results with significantly less residual PTT measured on posterior stress radiographs in patients undergoing isolated DB (*n* = 28) PCL-R compared to SB (*n* = 25) PCL-R [88]. However, the authors questioned the clinical relevance of the statistically significant difference of 1.4 mm between the groups [88]. Accordingly, no difference in clinical and radiological outcomes, failure and survival rates between SB (*n* = 28) and DB (*n* = 36) PCL-R could be observed in long-term follow-up (minimum 10 years) [89].

Recently, remnant preservation in SB and DB transtibial PCL-R has gained increasing interest. Preservation of the remnant PCL fibers and meniscofemoral ligaments is believed to provide graft protection by stabilizing the graft and additionally reducing the killer-turn effect by cushioning the graft at the proximal aperture of the tibial tunnel [38, 39, 78, 88]. Therefore, improved graft healing and maturation is assumed by enhanced revascularization and regeneration of mechanoreceptors [38, 39]. In one study, improved patient-reported outcomes, extensor and flexor muscle peak torque, functional performance, and decreased PTT, based on posterior sagittal stress radiographs, have been reported in 52 isolated SB transtibial PCL-R with remnant preservation after a mean follow-up duration of 30 months [39]. In this study cohort, acute PCL-R (mean time from injury to surgery, 2.4 months) enabled remnant preservation, which resulted in normal graft appearance (signal intensity, gross appearance) in almost 79% of patients as assessed by postoperative MRI (mean time from surgery to MRI, 15.8 months) [39]. Similar results have also been shown for combined PCL and posterolateral corner reconstruction [38].

### Tibial inlay vs. transtibial

In the early 1990s, a new technique for tibial graft fixation in PCL-R, termed the tibial inlay technique, was introduced [8, 27]. The tibial inlay technique was subsequently advocated to prevent increased graft stress, degeneration, and abrasion caused by the so-called ‘’killer turn’’ at the proximal tibial tunnel aperture in transtibial PCL-R [8]. However, a recently published study reported that remnant preservation in acute transtibial PCL-R enables to avoid the negative influence of the killer turn by a cushioning effect of the remnant PCL fibers [39]. Unfortunately, remnant preservation is not possible in chronic PCL deficiency and revision PCL-R, and, therefore, the tibial inlay technique has been suggested as a viable treatment alternative for such cases [41, 42, 59]. In spite of a biomechanically confirmed superiority of the tibial inlay technique compared to the transtibial technique in terms of residual posterior tibial laxity and graft degeneration [9], this is not translated into clinical outcomes [40, 50, 70, 75]. Since research has shown that there is no significant correlation between residual posterior tibial laxity and patient-reported outcomes, the biomechanically suggested superiority of the tibial inlay compared to the transtibial technique needs to be questioned [64, 73]. In one study, the tibial inlay technique was compared to the transtibial technique in 66 isolated PCL-Rs at a mean follow-up of 148 months [77]. Patients undergoing isolated tibial inlay PCL-R (*n* = 30) with bone-patellar tendon-bone autograft showed no difference in postoperative patient-reported outcomes (Lysholm Score and Tegner Activity Scale), manual laxity testing (posterior drawer test at 90° knee flexion), instrumented posterior laxity testing (stress radiographs at 90° knee flexion), and progression of OA compared to patients undergoing transtibial PCL-R (*n* = 36) with hamstring tendon autograft [77]. Consistently, most studies report statistically significant improvement in clinical and functional outcomes postoperatively compared to preoperatively [50, 70, 77]. However, regardless of the performed tibial fixation technique, considerable rates of residual posterior tibial laxity are reported. One comparative study has shown that 46% and 57% of patients undergoing transtibial and tibial inlay PCL-R, respectively, reported residual episodes of subjective instability [50]. On the other hand, the all-arthroscopic transtibial technique is surgically less demanding, avoids an invasive surgical approach, has a reduced operation time, has a lower risk of complications, and allows for the possibility for remnant preservation [40, 70]. However, the tibial inlay technique demonstrates advantages for the treatment of chronic PCL injuries and for the increasing number of revision PCL-Rs [8, 41, 42, 59]. Consequently, future research should focus on identifying specific indications for each technique and thus facilitate surgical decision-making.

### Primary vs. revision PCL-R

Failures after primary PCL-R are reported to cause severe impairments in daily living and are a burdening condition for most patients [41, 58, 59, 81]. In such cases, a revision PCL-R is required and is sometimes considered as a salvage procedure. The rate of revision surgery after isolated PCL-R is reported to be 3% and slightly higher (3.4%) after combined PCL-R [46]. However, only a few studies and case reports have reported on the outcomes of revision PCL-R [16, 41, 42, 47, 59, 81]. In one study, revision PCL-R using quadriceps tendon-bone autografts in a DB technique has significantly improved patient-reported outcomes, activities of daily living, sports activity level, occupational rate, and PTT based on posterior stress radiographs in 15 patients after a mean follow-up of 44 months [59]. Two of the subjects underwent revision PCL-Rs that failed a second time [59]. A recently published study has reported similar results after 22 revision PCL-Rs using Achilles tendon allografts with a DB tibial inlay technique [41]. Patient-reported outcomes and objective evaluation increased significantly while PTT, based on posterior stress radiographs as well as knee laxity (measured by KT-1000 arthrometry), decreased significantly after a mean follow-up of 40 months. Additionally, based on the Tegner Activity Scale, 77% of patients undergoing revision PCL-R were able to return to normal activities of daily living [41]. However, research has shown that 46% of failed PCL operative procedures are not amenable to revision PCL-R mostly due to advanced degenerative changes and OA [58]. Given that revision PCL-R is inherently related to an alteration of the native anatomy and the bony landmarks due to the primary PCL-R, revision surgeries represent more challenging procedures [59]. Therefore, it is recommended to perform a comprehensive diagnostic work-up including patient history, thorough clinical examination, gait analysis, AP, lateral, and weight-bearing hip-knee-ankle radiographs as well as MRI and CT scans prior to considering revision PCL-R. Consequently, risk factors related to failures in the operative treatment of PCL injuries can be assessed to facilitate treatment decision-making [58, 59].

## Failure analysis and survivorship in PCL-R

**Fig. 2:**
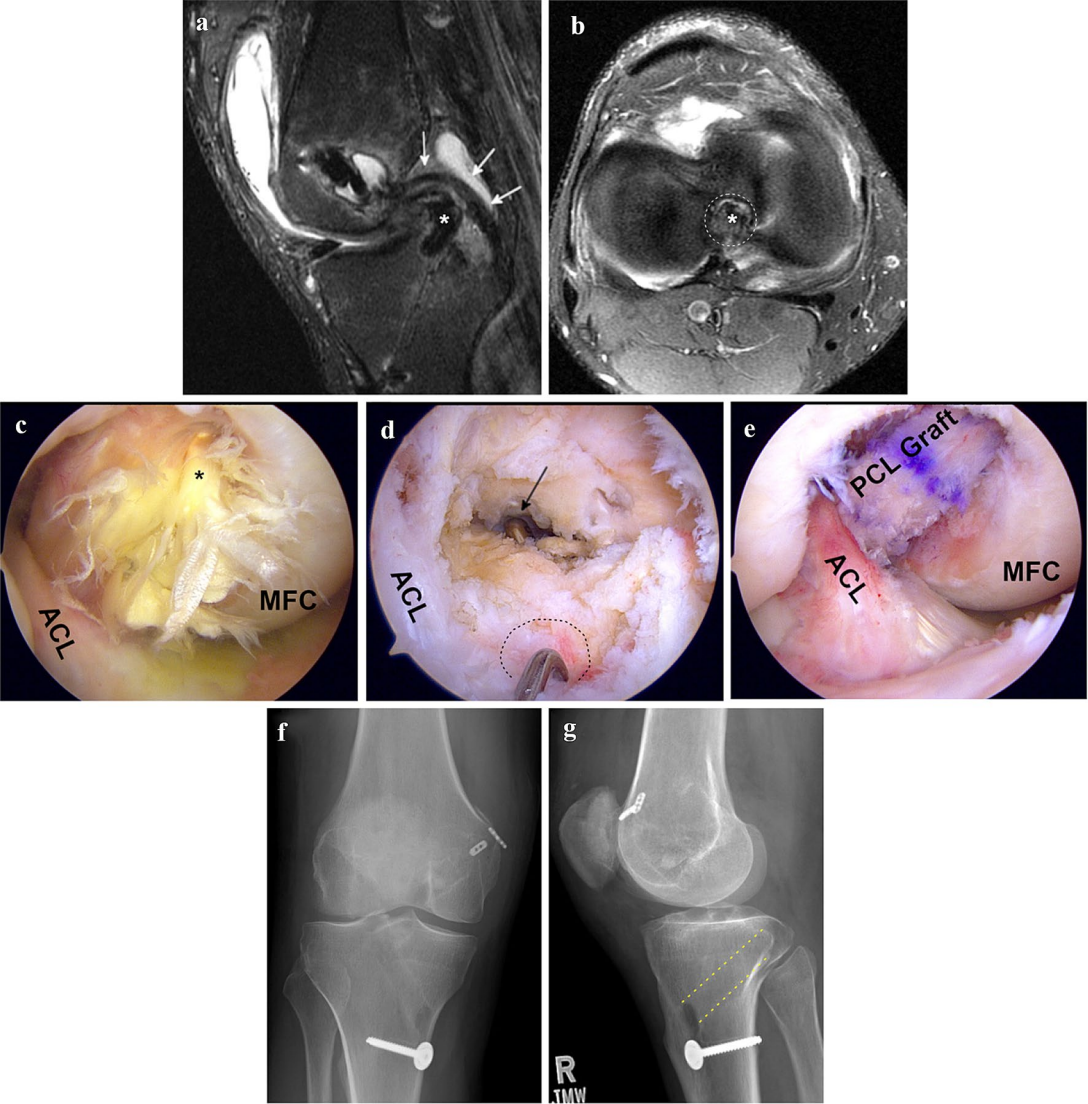
Posterior cruciate ligament graft failure. Patient with atraumatic PCL graft failure of the right knee. T2-weighted sagittal (**a**) and axial (**b**) MR images showing PCL graft failure and misplaced tibial tunnel (too anterior and too proximal). Note scarring of remnant PCL fibers imitating PCL continuity (white arrows). Arthroscopic images demonstrating graft failure (**c**), misplaced tibial tunnel (**d**), and revision PCL graft (**e**). Postoperative anterior–posterior (**f**) and lateral (**g**) radiographs demonstrating new anatomic tibial tunnel. white/black dashed lines, misplaced tibial tunnel; yellow dashed lines, new anatomic tibial tunnel; black arrow, new anatomic tibial tunnel during revision PCL reconstruction; *deficient PCL graft, *ACL* anterior cruciate ligament, *MFC* medial femoral condyle, *MR* magnetic resonance, *PCL* posterior cruciate ligament

Unlike in ACL reconstructive procedures, less is known about risk factors and causes of failure in the treatment of PCL injuries [16, 41, 58, 81]. Failed operative treatment of isolated and combined PCL injuries is associated with severe limitations in daily, professional, and sports activities [58, 59, 81]. One study investigating 52 failed operative PCL procedures (including SB PCL-R, PCL repair, synthetic graft replacement, and PCL thermoplasty) reported that 71% of patients complained of moderate to severe pain during daily activities, 49% described their own knee condition as poor, and 75% have completely quit sports activities after a mean time of 42 months after the failed operative PCL treatment [58]. However, synthetic graft replacement and thermoplasty as a treatment for PCL injuries have been abandoned, which needs to be considered when interpreting the reported poor results. To avoid the detrimental effects of failed PCL-R, it is essential to be aware of the risk factors and understand the underlying causes of failure. In more than 50% of failed PCL-R, multiple factors for failure have been identified [16, 41, 58]. Posterolateral corner deficiency and femoral or tibial tunnel misplacement have been shown to be the most common causes of failure, accounting for 40–77% and 33–41%, respectively (Fig. 2) [41, 58]. An incorrect tunnel placement is characterized by a too proximal (deep) and posterior (low) position on the femoral site or a too anterior and proximal position on the tibial site, resulting in a vertical position of the graft [56, 58]. Further risk factors are varus malalignment, primary PCL suture repair, biological and technical failures, wrong surgical decision-making, a too early or too progressive rehabilitation protocol, and a low annual volume of PCL-Rs performed by the operating surgeon [16, 58, 81].

Recently, a biomechanical study has shown an inverse correlation between the graft force after PCL-R and the posterior tibial slope (PTS). Irrespective of the loading condition and the knee flexion angle, a flatter (reduced) PTS leads to increasing PCL graft forces [10]. Clinically, a significant negative correlation between PTS and residual PTT measured on posterior stress radiographs and a significant positive correlation between PTS and the reduction of PTT from pre- to postoperative has been shown for SB PCL-R [22]. However, this observation has not been demonstrated for DB PCL-R [11]. Additionally, a significantly lower PTS has been observed in patients undergoing primary PCL-R compared to sex- and age-matched controls without ligamentous injury (6° vs. 9°) [12]. Analysis of the injury mechanism revealed that the PTS was significantly lower in non-contact PCL injuries compared to contact injuries (5° vs. 6°) [12], highlighting the impact of the PTS on AP laxity.

At 15 years follow-up with graft failure as the endpoint (need for revision PCL-R, high tibial osteotomy, arthroplasty, complete graft tear based on MRI, or > 10 mm side-to-side difference in PTT based on posterior stress radiographs), the survival rates have been reported to be approximately 82% and 84% for SB (*n* = 28) and DB (*n* = 36) Achilles tendon allograft PCL-R, respectively [89].

## Complications in PCL-R

Complications in isolated and combined PCL-R have been reported to occur in up to 53% of surgeries and can be divided into complications generally associated with operative procedures and in complications which are inherently related to the different techniques in PCL-R [15, 34, 36, 38, 40, 45, 59, 67, 70, 77, 81, 88, 90]. Well-known complications in knee surgery and thus also described in PCL-R are postoperative hematoma, surgical site infections, arthrofibrosis, reflex sympathetic dystrophy syndrome, anterior knee pain, paresthesia, neurovascular injuries, deep vein thrombosis, and graft failure with recurrent pain and instability [15, 16, 36, 59, 67, 70, 77, 81, 87, 90]. Iatrogenic injuries to the neurovascular structures of the posterior part of the knee represent the most dreaded complications in PCL-R. The occurrence is rare, yet several case reports about popliteal artery lacerations, occlusions, and also popliteal vein injuries have been published [51, 57, 86]. Precise anatomic knowledge and fluoroscopically guided tibial tunnel drilling in 90° knee flexion may help to prevent iatrogenic neurovascular injuries [33, 54]. In the setting of revision PCL-R, scar formation has to be considered, which may alter the natural course of the neurovascular structures.

A meta-analysis reported that the incidence of perioperative complications is 1.7 times higher for the tibial inlay compared to the transtibial technique. However, no statistically significant difference was noted [40]. The open tibial inlay technique requires careful dissection of the popliteal fossa and poses the risk of neurovascular injuries [8, 42, 81]. Consequently, all-arthroscopic tibial inlay techniques have evolved and have been shown to be biomechanically comparable to an open tibial inlay technique [91]. Additional reported complications related to the tibial inlay technique include fractures of the tibial bone plug during graft fixation [34]. Double-bundle PCL-R is surgically challenging and requires precise knowledge of anatomy and bony landmarks for accurate tunnel placement [5, 56]. One study reported a fracture of the separating bony bridge between the ALB and PMB femoral tunnel in DB PCL-R [87]. Furthermore, the tibial inlay and DB techniques are associated with a significantly longer operation time compared to the transtibial and SB techniques, increasing the overall risk of peri- and postoperative complications [45, 70].

## Non-operative treatment, rehabilitation, and return-to-sports (RTS)

Within the past 10 years (2010s), studies have consistently supported non-operative treatment for isolated grade I, grade II, and nondisplaced tibial avulsion PCL injuries [52, 63, 65, 71, 72]. There continues to be a debate regarding the management of isolated grade III injuries as there is limited data on the outcomes following non-operative treatment. A prospective cohort study in high-level athletes with grade II (*n* = 25) and grade III (*n* = 21) isolated acute PCL injuries showed that approximately 83% of athletes were able to participate at a competitive sports level (mean Tegner Activity Scale, 9) after non-operative treatment at an average follow-up period of 5 years [2]. In addition, an epidemiological study demonstrated a median lay-off time of 31 days after PCL injury for professional male soccer players. However, these prospectively collected data in men’s professional soccer included all grades of PCL injuries as well as operatively and non-operatively treated athletes [48]. Accordingly, initial non-operative management based on functional bracing and rehabilitation with optional delayed PCL-R seems to be reasonable for isolated acute PCL injuries, even for high-level athletes with grade III PCL injuries [2]. Although the PCL has a strong intrinsic healing capability, residual posterior laxity is a serious and frequently observed disadvantage of non-operative management [52, 71, 72]. However, the subjective and objective outcomes after non-operative treatment are promising [3, 26, 30, 63, 71, 72]. One prospective study demonstrated increased knee laxity based on manual testing in 9% of patients following non-operative treatment after a mean follow-up of 14 years. Additionally, instrumented laxity testing (KT-1000) revealed a mean side-to-side difference of 3 mm [71]. Nevertheless, the majority of patients were able to regain functional range-of-motion (ROM) and sufficient quadriceps strength to return to activities of daily living, with 45% participating in jumping and pivoting activities [71]. Furthermore, no correlation between functional outcomes and grade of laxity could be observed [71]. While non-operative management remains an integral part of the management of isolated PCL injures, it is important to acknowledge that unsatisfactory outcomes may occur. One study showed that patients undergoing non-operative treatment of isolated PCL injuries occasionally experienced pain and swelling in 81% and 56% of patients, respectively [13]. Additionally, a considerable number of PCL deficient patients developed subsequent meniscal injuries requiring subsequent surgery as well as a deterioration of the articular cartilage on average 13 years after the injury, indicating residual knee laxity [13]. This is also supported by the development of moderate to severe OA in approximately 11% of patients at long-term follow-up [71]. There is a paucity of studies comparing operative and non-operative treatment in PCL deficient patients. However, it has been shown that non-operative treatment leads to significantly more subsequent meniscal injuries as well as a higher rate of OA and a higher conversion rate to total knee arthroplasty compared to operative treatment [83].

Rehabilitation protocols whether for non-operative treatment or postoperative care, are inconsistently reported in the literature [65]. Agreement exists in the combination of temporary immobilization/bracing and exercise therapy. Accordingly, appropriate stabilization by initial static and later functional bracing accompanied by progressive exercise therapy is important, whether post-injury or postoperatively, to support the healing process of the PCL [3, 26, 30]. A dynamic anterior drawer brace facilitates end-to-end contact between the torn PCL fibers by applying an anteriorly directed force along the proximal tibia [37]. Studies demonstrated a reduction of PTT based on instrumented laxity measurement following non-operative treatment using static and dynamic braces with posterior tibial support [26, 30]. Initially, partial weight-bearing is recommended and ROM exercises are performed in the prone position to minimize hamstring activity and to counteract the gravity-induced posterior tibial sag [2, 35, 65]. The following weeks are accompanied by advancement to full weight-bearing with strong emphasis on quadriceps strengthening. Jogging and sport-specific exercises are often initiated in the sixth postoperative month. Full ROM, quadriceps strength, and a firm endpoint in the posterior drawer test are required before return to cutting and pivoting sports [35, 65]. This can take up to 12 months, however, quicker recovery with return to sports at 16 weeks has been reported in high-level athletes [2].

**Fig. 3:**
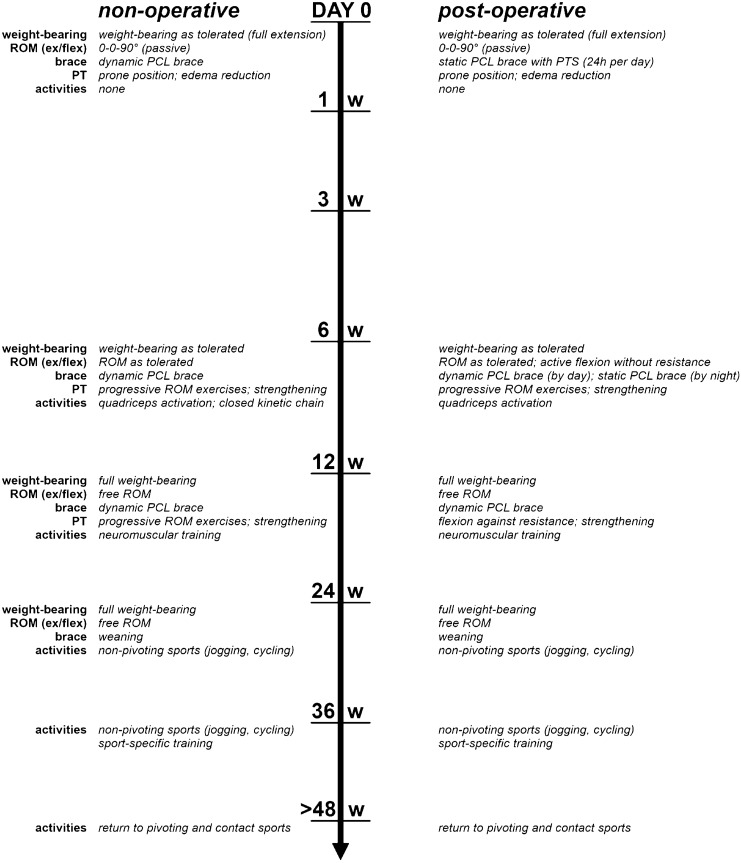
Non-operative and postoperative treatment protocol for posterior cruciate ligament injuries. *PCL* posterior cruciate ligament, *PT* physical therapy, *PTS* posterior tibial support, *ROM* *(ex/flex)* range of motion (extension to flexion), *w* week

Following PCL-R, weight-bearing as tolerated with a knee brace providing posterior tibial support and locked in full extension is recommended for the first 3–6 weeks, followed by functional bracing for up to 6 months, to promote healing and prevent a fixed posterior tibial subluxation [19, 35, 65]. The authors’ recommendation for non-operative treatment and postoperative rehabilitation is illustrated in Fig. 3.

## Future research

It is unknown which demographic, surgical, or patient-related factors reliably predict clinical and functional outcomes for both operative and non-operative treatment. Since non-operative treatment may yield satisfactory outcomes, an initial non-operative treatment of PCL injuries with an optional delayed PCL-R is often recommended. However, the optimal timing of operative PCL-R (early vs. delayed surgery) as well as the most appropriate timing of post-injury/postoperative rehabilitation (early vs. delayed) is not sufficiently supported by high-quality evidence, which is even more pronounced in combined PCL injuries. Therefore, a prospective randomized multi-center clinical trial—Surgical Timing and Rehabilitation (STaR) Trial for Multiple Ligament Knee Injuries—is currently ongoing to provide evidence for the optimal timing of operative treatment and non-operative/postoperative rehabilitation [49].

## Conclusion

As demonstrated in this two-part review PCL injuries are complex and commonly associated with neurovascular compromise and multiple ligament knee injuries involving the PLC and less commonly MCL. Clinicians must have a thorough understanding of anatomy and biomechanics to aid in decision-making, analysis of concomitant injuries, and operative treatment. Treatment algorithms following history and physical examination involve advanced imaging including stress radiographs and MRI. Exact treatment decisions are finalized and often revised after examination under anesthesia and afford availability of multiple surgical tools and graft choices as well as the flexibility of the experienced surgeon. Good to excellent outcomes with high patient satisfaction can be achieved. High-quality and large-scale studies are needed to provide further evidence for an individualized treatment approach pursuing the ultimate goal of restoring native knee kinematics and facilitating a return to daily, professional, and sports activities.

## Abbreviations

ACL: Anterior cruciate ligament
ALB: Anterolateral bundle
AP: Anterior–posterior
DB: Double-bundle
IKDC: International Knee Documentation Committee
MCL: Medial collateral ligament
OA: Osteoarthritis
PCL: Posterior cruciate ligament
PCL-R: Posterior cruciate ligament reconstruction
PMB: Posteromedial bundle
PMC: Posteromedial corner
PTS: Posterior tibial slope
PTT: Posterior tibial translation
ROM: Range of motion
RTS: Return-to-sports
SB: Single-bundle

## References

[1] Aglietti P, Giron F, Losco M, Cuomo P, Ciardullo A, Mondanelli N (2010). Comparison between single-and double-bundle anterior cruciate ligament reconstruction: a prospective, randomized, single-blinded clinical trial. Am J Sports Med.

[2] Agolley D, Gabr A, Benjamin-Laing H, Haddad FS (2017). Successful return to sports in athletes following non-operative management of acute isolated posterior cruciate ligament injuries: medium-term follow-up. Bone Jt J.

[3] Ahn JH, Lee SH, Choi SH, Wang JH, Jang SW (2011). Evaluation of clinical and magnetic resonance imaging results after treatment with casting and bracing for the acutely injured posterior cruciate ligament. Arthroscopy.

[4] Ahrend M, Ateschrang A, Döbele S, Stöckle U, Grünwald L, Schröter S (2016). Return to sport after surgical treatment of a posterior cruciate ligament injury: a retrospective study of 60 patients. Orthopade.

[5] Anderson CJ, Ziegler CG, Wijdicks CA, Engebretsen L, LaPrade RF (2012). Arthroscopically pertinent anatomy of the anterolateral and posteromedial bundles of the posterior cruciate ligament. J Bone Jt Surg Am.

[6] Ansari AS, Dennis BB, Horner NS, Zhu M, Brookes C, Khan M (2019). Influence of graft source on postoperative activity and joint laxity in posterior cruciate ligament reconstruction: a systematic review. Arthroscopy.

[7] Belk JW, Kraeutler MJ, Purcell JM, McCarty EC (2018). Autograft versus allograft for posterior cruciate ligament reconstruction: an updated systematic review and meta-analysis. Am J Sports Med.

[8] Berg EE (1995). Posterior cruciate ligament tibial inlay reconstruction. Arthroscopy.

[9] Bergfeld JA, McAllister DR, Parker RD, Valdevit AD, Kambic HE (2001). A biomechanical comparison of posterior cruciate ligament reconstruction techniques. Am J Sports Med.

[10] Bernhardson AS, Aman ZS, DePhillipo NN, Dornan GJ, Storaci HW, Brady AW (2019). Tibial slope and its effect on graft force in posterior cruciate ligament reconstructions. Am J Sports Med.

[11] Bernhardson AS, DePhillipo NN, Aman ZS, Kennedy MI, Dornan GJ, LaPrade RF (2019). Decreased posterior tibial slope does not affect postoperative posterior knee laxity after double-bundle posterior cruciate ligament reconstruction. Am J Sports Med.

[12] Bernhardson AS, DePhillipo NN, Daney BT, Kennedy MI, Aman ZS, LaPrade RF (2019). Posterior tibial slope and risk of posterior cruciate ligament injury. Am J Sports Med.

[13] Boynton MD, Tietjens BR (1996). Long-term followup of the untreated isolated posterior cruciate ligament-deficient knee. Am J Sports Med.

[14] Campbell RB, Torrie A, Hecker A, Sekiya JK (2007). Comparison of tibial graft fixation between simulated arthroscopic and open inlay techniques for posterior cruciate ligament reconstruction. Am J Sports Med.

[15] Chahla J, Moatshe G, Cinque ME, Dornan GJ, Mitchell JJ, Ridley TJ (2017). Single-bundle and double-bundle posterior cruciate ligament reconstructions: a systematic review and meta-analysis of 441 patients at a minimum 2 years' follow-up. Arthroscopy.

[16] Cooper DE, Stewart D (2004). Posterior cruciate ligament reconstruction using single-bundle patella tendon graft with tibial inlay fixation: 2- to 10-year follow-up. Am J Sports Med.

[17] Devitt BM, Dissanayake R, Clair J, Napier RJ, Porter TJ, Feller JA (2018). Isolated posterior cruciate reconstruction results in improved functional outcome but low rates of return to preinjury level of sport: a systematic review and meta-analysis. Orthop J Sports Med.

[18] Fanelli GC (2020). PCL transtibial tunnel reconstruction. Sports Med Arthrosc Rev.

[19] Fanelli GC (2008). Posterior cruciate ligament rehabilitation: how slow should we go?. Arthroscopy.

[20] Grassmayr MJ, Parker DA, Coolican MR, Vanwanseele B (2008). Posterior cruciate ligament deficiency: biomechanical and biological consequences and the outcomes of conservative treatment. A systematic review. J Sci Med Sport.

[21] Gupta A, Lattermann C, Busam M, Riff A, Bach BR, Wang VM (2009). Biomechanical evaluation of bioabsorbable versus metallic screws for posterior cruciate ligament inlay graft fixation: a comparative study. Am J Sports Med.

[22] Gwinner C, Weiler A, Roider M, Schaefer FM, Jung TM (2017). Tibial slope strongly influences knee stability after posterior cruciate ligament reconstruction: a prospective 5- to 15-year follow-up. Am J Sports Med.

[23] Harner CD, Janaushek MA, Kanamori A, Yagi M, Vogrin TM, Woo SL (2000). Biomechanical analysis of a double-bundle posterior cruciate ligament reconstruction. Am J Sports Med.

[24] Harner CD, Janaushek MA, Ma CB, Kanamori A, Vogrin TM, Woo SL (2000). The effect of knee flexion angle and application of an anterior tibial load at the time of graft fixation on the biomechanics of a posterior cruciate ligament-reconstructed knee. Am J Sports Med.

[25] Hudgens JL, Gillette BP, Krych AJ, Stuart MJ, May JH, Levy BA (2013). Allograft versus autograft in posterior cruciate ligament reconstruction: an evidence-based systematic review. J Knee Surg.

[26] Jacobi M, Reischl N, Wahl P, Gautier E, Jakob RP (2010). Acute isolated injury of the posterior cruciate ligament treated by a dynamic anterior drawer brace: a preliminary report. J Bone Jt Surg Br.

[27] Jakob RP, Rüegsegger M (1993). Therapy of posterior and posterolateral knee instability. Orthopade.

[28] Johannsen AM, Anderson CJ, Wijdicks CA, Engebretsen L, LaPrade RF (2013). Radiographic landmarks for tunnel positioning in posterior cruciate ligament reconstructions. Am J Sports Med.

[29] Jung TM, Reinhardt C, Scheffler SU, Weiler A (2006). Stress radiography to measure posterior cruciate ligament insufficiency: a comparison of five different techniques. Knee Surg Sports Traumatol Arthrosc.

[30] Jung YB, Tae SK, Lee YS, Jung HJ, Nam CH, Park SJ (2008). Active non-operative treatment of acute isolated posterior cruciate ligament injury with cylinder cast immobilization. Knee Surg Sports Traumatol Arthrosc.

[31] Kennedy NI, LaPrade RF, Goldsmith MT, Faucett SC, Rasmussen MT, Coatney GA (2014). Posterior cruciate ligament graft fixation angles, part 1: biomechanical evaluation for anatomic single-bundle reconstruction. Am J Sports Med.

[32] Kennedy NI, LaPrade RF, Goldsmith MT, Faucett SC, Rasmussen MT, Coatney GA (2014). Posterior cruciate ligament graft fixation angles, part 2: biomechanical evaluation for anatomic double-bundle reconstruction. Am J Sports Med.

[33] Kim J, Allaire R, Harner CD (2010). Vascular safety during high tibial osteotomy: a cadaveric angiographic study. Am J Sports Med.

[34] Kim SJ, Kim TE, Jo SB, Kung YP (2009). Comparison of the clinical results of three posterior cruciate ligament reconstruction techniques. J Bone Jt Surg Am.

[35] Klose M, Forkel P, Imhoff A (2018). Rehabilitation after reconstruction of the posterior cruciate ligament. MMW Fortschr Med.

[36] LaPrade RF, Cinque ME, Dornan GJ, DePhillipo NN, Geeslin AG, Moatshe G (2018). Double-bundle posterior cruciate ligament reconstruction in 100 patients at a mean 3 years' follow-up: outcomes were comparable to anterior cruciate ligament reconstructions. Am J Sports Med.

[37] LaPrade RF, Smith SD, Wilson KJ, Wijdicks CA (2015). Quantification of functional brace forces for posterior cruciate ligament injuries on the knee joint: an in vivo investigation. Knee Surg Sports Traumatol Arthrosc.

[38] Lee DW, Jang HW, Lee YS, Oh SJ, Kim JY, Song HE (2014). Clinical, functional, and morphological evaluations of posterior cruciate ligament reconstruction with remnant preservation: minimum 2-year follow-up. Am J Sports Med.

[39] Lee DW, Kim JG, Yang SJ, Cho SI (2019). Return to sports and clinical outcomes after arthroscopic anatomic posterior cruciate ligament reconstruction with remnant preservation. Arthroscopy.

[40] Lee DY, Kim DH, Kim HJ, Ahn HS, Lee TH, Hwang SC (2018). Posterior cruciate ligament reconstruction with transtibial or tibial inlay techniques: a meta-analysis of biomechanical and clinical outcomes. Am J Sports Med.

[41] Lee SH, Jung YB, Lee HJ, Jung HJ, Kim SH (2012). Revision posterior cruciate ligament reconstruction using a modified tibial-inlay double-bundle technique. J Bone Jt Surg Am.

[42] Lee SH, Jung YB, Rhee SM, Lee HJ, Jung HJ (2014). Revision posterior cruciate ligament reconstruction with a modified tibial-inlay double-bundle technique. JBJS Essent Surg Tech.

[43] Li G, Papannagari R, Li M, Bingham J, Nha KW, Allred D (2008). Effect of posterior cruciate ligament deficiency on in vivo translation and rotation of the knee during weightbearing flexion. Am J Sports Med.

[44] Li J, Kong F, Gao X, Shen Y, Gao S (2016). Prospective randomized comparison of knee stability and proprioception for posterior cruciate ligament reconstruction with autograft, hybrid graft, and γ-irradiated allograft. Arthroscopy.

[45] Li Y, Li J, Wang J, Gao S, Zhang Y (2014). Comparison of single-bundle and double-bundle isolated posterior cruciate ligament reconstruction with allograft: a prospective, randomized study. Arthroscopy.

[46] Lind M, Nielsen TG, Behrndtz K (2018). Both isolated and multi-ligament posterior cruciate ligament reconstruction results in improved subjective outcome: results from the Danish Knee Ligament Reconstruction Registry. Knee Surg Sports Traumatol Arthrosc.

[47] Lubis AMT, Kuncoro MW (2019). Revision of failed-posterior cruciate ligament (PCL) reconstruction due to tibial tunnel misplacement: a case report. Ann Med Surg (Lond).

[48] Lundblad M, Hägglund M, Thomeé C, Hamrin Senorski E, Ekstrand J, Karlsson J (2020). Epidemiological data on LCL and PCL injuries over 17 seasons in men's professional soccer: the UEFA elite club injury study. Open Access J Sports Med.

[49] Lynch AD, Chmielewski T, Bailey L, Stuart M, Cooper J, Coady C (2017). Current concepts and controversies in rehabilitation after surgery for multiple ligament knee injury. Curr Rev Musculoskelet Med.

[50] MacGillivray JD, Stein BE, Park M, Allen AA, Wickiewicz TL, Warren RF (2006). Comparison of tibial inlay versus transtibial techniques for isolated posterior cruciate ligament reconstruction: minimum 2-year follow-up. Arthroscopy.

[51] Makino A, Costa-Paz M, Aponte-Tinao L, Ayerza MA, Muscolo DL (2005). Popliteal artery laceration during arthroscopic posterior cruciate ligament reconstruction. Arthroscopy.

[52] Mariani PP, Margheritini F, Christel P, Bellelli A (2005). Evaluation of posterior cruciate ligament healing: a study using magnetic resonance imaging and stress radiography. Arthroscopy.

[53] Markolf KL, Zemanovic JR, McAllister DR (2002). Cyclic loading of posterior cruciate ligament replacements fixed with tibial tunnel and tibial inlay methods. J Bone Jt Surg Am.

[54] Marom N, Ruzbarsky JJ, Boyle C, Marx RG (2020). Complications in posterior cruciate ligament injuries and related surgery. Sports Med Arthrosc Rev.

[55] Montgomery SR, Johnson JS, McAllister DR, Petrigliano FA (2013). Surgical management of PCL injuries: indications, techniques, and outcomes. Curr Rev Musculoskelet Med.

[56] Narvy SJ, Pearl M, Vrla M, Yi A, Hatch GF (2015). Anatomy of the femoral footprint of the posterior cruciate ligament: a systematic review. Arthroscopy.

[57] Nemani VM, Frank RM, Reinhardt KR, Pascual-Garrido C, Yanke AB, Drakos M (2012). Popliteal venotomy during posterior cruciate ligament reconstruction in the setting of a popliteal artery bypass graft. Arthroscopy.

[58] Noyes FR, Barber-Westin SD (2005). Posterior cruciate ligament revision reconstruction, part 1: causes of surgical failure in 52 consecutive operations. Am J Sports Med.

[59] Noyes FR, Barber-Westin SD (2005). Posterior cruciate ligament revision reconstruction, part 2: results of revision using a 2-strand quadriceps tendon-patellar bone autograft. Am J Sports Med.

[60] Nuelle CW, Milles JL, Pfeiffer FM, Stannard JP, Smith PA, Kfuri M (2017). Biomechanical comparison of five posterior cruciate ligament reconstruction techniques. J Knee Surg.

[61] Owesen C, Sandven-Thrane S, Lind M, Forssblad M, Granan LP, Årøen A (2017). Epidemiology of surgically treated posterior cruciate ligament injuries in Scandinavia. Knee Surg Sports Traumatol Arthrosc.

[62] Panchal HB, Sekiya JK (2011). Open tibial inlay versus arthroscopic transtibial posterior cruciate ligament reconstructions. Arthroscopy.

[63] Parolie JM, Bergfeld JA (1986). Long-term results of nonoperative treatment of isolated posterior cruciate ligament injuries in the athlete. Am J Sports Med.

[64] Patel DV, Allen AA, Warren RF, Wickiewicz TL, Simonian PT (2007). The nonoperative treatment of acute, isolated (partial or complete) posterior cruciate ligament-deficient knees: an intermediate-term follow-up study. HSS J.

[65] Pierce CM, O'Brien L, Griffin LW, Laprade RF (2013). Posterior cruciate ligament tears: functional and postoperative rehabilitation. Knee Surg Sports Traumatol Arthrosc.

[66] Race A, Amis AA (1998). PCL reconstruction. In vitro biomechanical comparison of 'isometric' versus single and double-bundled 'anatomic' grafts. J Bone Jt Surg Br.

[67] Rhatomy S, Abadi MBT, Setyawan R, Asikin AIZ, Soekarno NR, Imelda LG (2020). Posterior cruciate ligament reconstruction with peroneus longus tendon versus hamstring tendon: a comparison of functional outcome and donor site morbidity. Knee Surg Sports Traumatol Arthrosc.

[68] Schulz MS, Steenlage ES, Russe K, Strobel MJ (2007). Distribution of posterior tibial displacement in knees with posterior cruciate ligament tears. J Bone Jt Surg Am.

[69] Sekiya JK, Whiddon DR, Zehms CT, Miller MD (2008). A clinically relevant assessment of posterior cruciate ligament and posterolateral corner injuries. Evaluation of isolated and combined deficiency. J Bone Jt Surg Am.

[70] Seon JK, Song EK (2006). Reconstruction of isolated posterior cruciate ligament injuries: a clinical comparison of the transtibial and tibial inlay techniques. Arthroscopy.

[71] Shelbourne KD, Clark M, Gray T (2013). Minimum 10-year follow-up of patients after an acute, isolated posterior cruciate ligament injury treated nonoperatively. Am J Sports Med.

[72] Shelbourne KD, Davis TJ, Patel DV (1999). The natural history of acute, isolated, nonoperatively treated posterior cruciate ligament injuries. A prospective study. Am J Sports Med.

[73] Shelbourne KD, Muthukaruppan Y (2005). Subjective results of nonoperatively treated, acute, isolated posterior cruciate ligament injuries. Arthroscopy.

[74] Shin J, Maak TG (2018). Arthroscopic transtibial PCL reconstruction: surgical technique and clinical outcomes. Curr Rev Musculoskelet Med.

[75] Shin YS, Kim HJ, Lee DH (2017). No clinically important difference in knee scores or instability between transtibial and inlay techniques for PCL reconstruction: a systematic review. Clin Orthop Relat Res.

[76] Shon OJ, Lee DC, Park CH, Kim WH, Jung KA (2010). A comparison of arthroscopically assisted single and double bundle tibial inlay reconstruction for isolated posterior cruciate ligament injury. Clin Orthop Surg.

[77] Song EK, Park HW, Ahn YS, Seon JK (2014). Transtibial versus tibial inlay techniques for posterior cruciate ligament reconstruction: long-term follow-up study. Am J Sports Med.

[78] Song JG, Kim HJ, Han JH, Bhandare NN, Shetty GM, Kang SB (2015). Clinical outcome of posterior cruciate ligament reconstruction with and without remnant preservation. Arthroscopy.

[79] Spiridonov SI, Slinkard NJ, LaPrade RF (2011). Isolated and combined grade-III posterior cruciate ligament tears treated with double-bundle reconstruction with use of endoscopically placed femoral tunnels and grafts: operative technique and clinical outcomes. J Bone Jt Surg Am.

[80] Sun X, Zhang J, Qu X, Zheng Y (2015). Arthroscopic posterior cruciate ligament reconstruction with allograft versus autograft. Arch Med Sci.

[81] Tucker CJ, Cotter EJ, Waterman BR, Kilcoyne KG, Cameron KL, Owens BD (2019). Functional outcomes after isolated and combined posterior cruciate ligament reconstruction in a military population. Orthop J Sports Med.

[82] Wang CJ, Chan YS, Weng LH, Yuan LJ, Chen HS (2004). Comparison of autogenous and allogenous posterior cruciate ligament reconstructions of the knee. Injury.

[83] Wang SH, Chien WC, Chung CH, Wang YC, Lin LC, Pan RY (2018). Long-term results of posterior cruciate ligament tear with or without reconstruction: a nationwide, population-based cohort study. PLoS ONE.

[84] Weimann A, Wolfert A, Zantop T, Eggers AK, Raschke M, Petersen W (2007). Reducing the "killer turn" in posterior cruciate ligament reconstruction by fixation level and smoothing the tibial aperture. Arthroscopy.

[85] Wijdicks CA, Kennedy NI, Goldsmith MT, Devitt BM, Michalski MP, Årøen A (2013). Kinematic analysis of the posterior cruciate ligament, part 2: a comparison of anatomic single- versus double-bundle reconstruction. Am J Sports Med.

[86] Wu RW, Hsu CC, Wang CJ (2003). Acute popliteal artery occlusion after arthroscopic posterior cruciate ligament reconstruction. Arthroscopy.

[87] Xu M, Zhang Q, Dai S, Teng X, Liu Y, Ma Z (2019). Double bundle versus single bundle reconstruction in the treatment of posterior cruciate ligament injury: a prospective comparative study. Indian J Orthop.

[88] Yoon KH, Bae DK, Song SJ, Cho HJ, Lee JH (2011). A prospective randomized study comparing arthroscopic single-bundle and double-bundle posterior cruciate ligament reconstructions preserving remnant fibers. Am J Sports Med.

[89] Yoon KH, Kim EJ, Kwon YB, Kim SG (2019). Minimum 10-year results of single- versus double-bundle posterior cruciate ligament reconstruction: clinical, radiologic, and survivorship outcomes. Am J Sports Med.

[90] Zayni R, Hager JP, Archbold P, Fournier Y, Quelard B, Chambat P (2011). Activity level recovery after arthroscopic PCL reconstruction: a series of 21 patients with a mean follow-up of 29 months. Knee.

[91] Zehms CT, Whiddon DR, Miller MD, Quinby JS, Montgomery SL, Campbell RB (2008). Comparison of a double bundle arthroscopic inlay and open inlay posterior cruciate ligament reconstruction using clinically relevant tools: a cadaveric study. Arthroscopy.

